# Stress of Conscience Questionnaire (SCQ): exploring dimensionality and psychometric properties at a tertiary hospital in Australia

**DOI:** 10.1186/s40359-020-00477-3

**Published:** 2020-10-20

**Authors:** Yangama Jokwiro, Elizabeth Pascoe, Kristina Edvardsson, Muhammad Aziz Rahman, Ewan McDonald, Qarin Lood, David Edvardsson

**Affiliations:** 1 College of Science, Health and Engineering, School of Nursing and Midwifery, La Trobe University, Melbourne, Australia; 2 School of Nursing and Healthcare Professions, Federation University, Berwick Campus, Melbourne, Australia; 3 Institute of Neuroscience and Physiology, Department of Health and Rehabilitation, Sahlgrenska Academy, Centre for Ageing and Health – AgeCap, University of Gothenburg, Box 455, 40530 Gothenburg, Sweden; 4 Department of Nursing, The Medical Faculty, Umea University, Umeå, Sweden; 5 School of Nursing and Midwifery, College of Science, Health and Engineering, La Trobe University, Bundoora, Australia

**Keywords:** Stress of conscience, Psychometrics, Dimensionality, Exploratory factor analysis and confirmatory factor analysis, Health professionals

## Abstract

**Background:**

This study explored the psychometric properties and dimensionality of the Stress of Conscience Questionnaire (SCQ) in a sample of health professionals from a tertiary-level Australian hospital. The SCQ, a measure of stress of conscience, is a recently developed nine-item instrument for assessing frequently encountered stressful situations in health care, and the degree to which they trouble the conscience of health professionals. This is relevant because stress of conscience has been associated with negative experiences such as job strain and/or burnout. The validity of SCQ has not been explored beyond Scandinavian contexts.

**Methods:**

A cross-sectional study of 253 health professionals was undertaken in 2015. The analysis involved estimates of reliability, variability and dimensionality. Exploratory factor analysis (EFA) and confirmatory factor analysis (CFA) were used to explore dimensionality and theoretical model fit respectively.

**Results:**

Cronbach’s alpha of 0.84 showed internal consistency reliability. All individual items of the SCQ (N = 9) met the cut-off criteria for item-total correlations (> 0.3) indicating acceptable homogeneity. Adequate variability was confirmed for most of the items, with some items indicating floor or ceiling effects. EFA retained a single latent factor with adequate factor loadings for a unidimensional structure. When the two‐factor model was compared to the one‐factor model, the latter achieved better goodness of fit supporting a one-factor model for the SCQ.

**Conclusion:**

The SCQ, as a unidimensional measure of stress of conscience, achieved adequate reliability and variability in this study. Due to unidimensionality of the tool, summation of a total score can be a meaningful way forward to summarise and communicate results from future studies, enabling international comparisons. However, further exploration of the questionnaire in other cultures and clinical settings is recommended to explore the stability of the latent one-factor structure.

## Background

The term ‘stress of conscience’ has emerged to conceptualise an existential dimension of stress health professionals may develop from frequently encountered stressful situations in health care, perceived as leading to a troubled conscience [1–4]. Despite the heterogeneity of clinical settings, the generic sources of frequently encountered stressful situations across health care settings include perceived demanding workload, lack of support from leadership/management and staff conflict [3, 4]. In such situations, health professionals perceived a gap between the reality of practice and their ideal practice, between structural demands and their own aspirations to provide the quality care they feel the person in need of care deserves [3–6]. Glasberg et al. [3] found that health professionals, reflecting on these stressful situations, often described punitive feelings of guilt, embarrassment and/or shame accompanying an experience of a troubled conscience. The extent to which they experienced a troubled conscience depended on individual appraisal of the stressful situation, which in turn is thought to be influenced by personal and professional ethical beliefs [1, 3–5]. Hence, Glasberg et al. [3] coined the term ‘stress of conscience’, to highlight and explore this existential dimension of workplace stress for health professionals.

The Stress of Conscience Questionnaire (SCQ) was developed to explore stress of conscience among health professionals [3]. The impetus for Glasberg and colleagues [3] to develop the SCQ came from their review of literature, which identified a gap in tools for assessing the phenomenon associated with everyday stressful workplace situations in which health professionals perceived that their actions or inactions contradicted their conscience. In addition, ethical studies in health care linked failure to heed the voice of conscience with negative workplace outcomes such as health professionals distancing themselves from persons in need of care clients, experiencing burnout, ill-health and staff attrition [7–11]. Based on these findings, Glasberg et al. [3] developed the SCQ and hypothesised that stress of conscience could be used as an early predictor of such negative workplace outcomes. This hypothesis received empirical support in recent Scandinavian studies which explored stress of conscience in a clinical setting using the SCQ [12–15]. Therein, high levels of stress of conscience positively correlated with ratings of burnout and job strain, while negatively correlating with job satisfaction [12, 13, 15]. Consequently, the SCQ could be a useful tool for detecting and understanding when health professionals feel stressed and are potentially on a detrimental path towards burnout and attrition. As such, the SCQ is worthy of further scrutiny beyond the Scandinavian context where the questionnaire has been validated.

The Stress of Conscience Questionnaire (SCQ), is a nine-item instrument for assessing stressful situations and the degree to which they cause a troubled conscience for health professionals [3]. This questionnaire asks respondents to first rate the frequency of which he/she perceives nine commonly occurring stressful situations present in their clinical setting on a scale of 0 ‘Never’ to 5 ‘Every day’ [3, 4]. Secondly, questionnaire asks respondents to rate the individual extent to which these situations are perceived as leading to a troubled conscience, on a scale from ‘not at all’ to ‘a very troubled conscience’ [3, 4]. The initial validation of the SCQ, identified two latent factors: ‘internal demands’ (Factor I) of the workplace, and ‘external demands and restrictions’ (Factor II) from sociocultural and religious beliefs [3]. Although two factors were identified, most studies have elected to present and interpret the result of the SCQ as a total sum score of all items ranging from 0 to 225 without the subscales [6, 12, 13]. Confidence in the utility of the subscales is yet to be established.

In terms of reliability, the SCQ was found to be reliable (Cronbach’s α = 0.83) in a Swedish sample of hospital staff, as well as in samples of staff from municipal and community health care centres [2, 3, 16]. In terms of dimensionality, although the initial SCQ validation indicated two latent factors (Factor I = 1nternal Demands and Factor II = External Demands and Restrictions), there were high cross-loadings for Items 1, 3 and 8 on both factors [3]. The final factor structure included Item 1 ‘*How often do you lack time to provide care that the patients’ needs*?’ in both published latent factor structures [3]. The re-validation by Ahlin et al. [4] also retained two latent factors, albeit these were different to the original theoretical interpretation, and a new interpretation was not provided. Instead, Ahlin et al. [4] suggested that the SCQ could be regarded as unidimensional after exclusion of Item 6 ‘*Is your private life ever so demanding that you don’t have the energy to devote yourself to your work as you would like*’. Furthermore, a study with a Finnish sample also retained two latent factors, which were inconsistent with the initial validation, and the factor outcomes were not theoretically interpreted [17]. It is plausible to conclude that, from the studies above, dimensionality of the questionnaire is yet to be settled, which warrants further exploration of the questionnaire in other contexts. Indeed, Glasberg et al. [3] and Ahlin et al. [4] also recommended exploration of the SCQ in other clinical settings, professions and cultural context.

To conclude, it seems pertinent to further explore the psychometric properties and dimensionality of the SCQ within an Australian context to provide further scrutiny beyond Scandinavian contexts. Findings could provide data and confidence (if upheld) to collect and compare results from the SCQ scale internationally.

## Aim of the study

This study aimed to explore psychometric properties and dimensionality of the SCQ in a sample of health professionals from a tertiary level hospital in Melbourne, Australia.

## Methods

### Study sample

The study was conducted in a sample of 253 nurses, medical doctors and allied health professionals across emergency, medical and surgical wards and a geriatric ward in a 560-bed Australian tertiary-level hospital. Administrative staff and other auxiliary staff members were excluded. A total of 500 questionnaires were distributed and 253 questionnaires where returned (51%).

### Stress of conscience questionnaire (SCQ)

The English version of the SCQ presented by Ahlin et al. [4] was used in this study. The questionnaire achieved a Cronbach’s alpha of 0.83 for the nine-item total validation in a Swedish context [4]. The SCQ is composed of nine two-part items (Part A and Part B) measuring commonly occurring stressful situations present in their clinical setting and the extent these situations are perceived as leading to a troubled conscience [22]. Part A assesses the frequency of such situations on a six-point Likert scale ranging from 0 (never) to 5 (every day). Part B assesses the extent to which these situations are perceived as leading to troubled conscience, on a visual analogue scale that runs from 0 (no, it does not trouble my conscience at all) to 5 (yes, it troubles my conscience greatly). The SCQ individual item score (index score) is obtained by multiplying part A and B ratings to generate a range from 0 to 25 points.

### Study procedure

The questionnaires which included demographic data such as age, sex and experience were delivered to the wards in a box that was stored in the nurse unit manager’s office, together with a sealed return box. The questionnaires were handed out to the staff during ward hand over. A participant information letter, which outlined the purpose of the study and guaranteed anonymity, accompanied each questionnaire. Participants were informed in the letter that consent was implied if they voluntarily completed and returned the questionnaire. All data were collected in October 2015 from voluntary participants.

### Statistical analysis

Questionnaire variability was analysed in terms of floor and ceiling effects, and a cut off score of > 15% on the minimum and maximum scores for each item was set [18]. Internal consistency reliability was evaluated by Cronbach’s alpha (> 0.7), item-total correlations (> 0.3) and inter-item correlations (0.2–0.4) [19, 20]. Exploratory factor analysis (EFA) was used to investigate instrument dimensionality. Kaiser-Meyer Olkin measure of sampling adequacy (KMO) and Bartlett’s test of sphericity were analysed first to determine suitability of the data to undergo factor analysis, the cut of were > 0.6 and < 1.0 and statistical significance (p < 0.001) respectively [21–23]. Confirmatory factor analysis (CFA) was used to examine the adequacy of the resulting factor model. To evaluate model fit, this study used a range of absolute and incremental model fit indices, including the ratio of chi-square to degrees of freedom (X2/df), comparative fit index (CFI), adjusted goodness of fit (AGFI); root mean square error of approximation (RMSEA), PCLOSE and Akaike Information Criteria (AIC), [24–26]. The factor structure in this study was also compared to the two-factor model proposed by Glasberg et al. [3]. The Statistical Package for Social Sciences (SPSS) and AMOS, Version 24.0 was used for statistical analysis of the data (SPSS, Chicago, IL, USA).

### Ethical considerations

The study adhered to the principles of the Helsinki Declaration and the National Health and Medical Research Council’s statement for the ethical conduct in human research. The study was approved by the Human Research Ethics Committee (LNR15, 299) to use implied informed consent, which meant that consent was obtained from participants if and when they returned a completed study questionnaire after reading the information letter which outlined the process. The reasoning behind this was to protect participant anonymity, privacy and autonomy, as far as possible by distributing study questionnaires at ward levels, making sure the informed consent to participate was made actively, individually and independently by those staff that completed and returned study questionnaires. This means that informed consent was implied in their active, autonomous and anonymous decision to participate.

## Results

### Sample characteristics

The sample consisted predominantly of registered nurses (n = 205, 81%), who were female (n = 217, 85.8%) as indicated in Table 1. The mean age was 32.9 (SD = 10.0) and the average length of time working in the ward was 9.2 years (SD = 9.0). The employment status was divided almost equally between full time (51.1%) and part-time/casual workers (48.9%). The specialty areas from which the sample was drawn are indicated in Table 1.

**Table 1:** Sample Characteristics

	*N* (%)
*Gender* (*n* = 253)	
Women	217 (85.8)
Men	36 (14.2)
*Qualification* (*n* = 253)	
Registered Nurses	205 (81)
Enrolled nurses	18 (7.1)
Medical Doctors	10 (4.0)
Physiotherapists	6 (2.4)
Occupational therapists	2 (0.8)
Others	12 (4.7)
*Age Group* (*n* = 253)	
20–29 years	126 (49.8)
30–39 years	72 (28.5)
40–49 years	24 (9.4)
50 +	31 (12.3)
*Specialty area (n* = 253)	
Emergency ward	59 (23.3)
Surgical wards	80 (31.6)
Medical wards	83 (32.8)
Geriatric ward	31 (12.3)

### Variability

Table 2 summarises item performance of the SCQ. The table presents Part A and Part B of the questions separately and as index scores (Part A multiplied by Part B). Ceiling effects were detected for Items 1A and 3A (39.4% and 17.4%) and a floor effect was detected in Items 2A (40.9%), 4A and 4B (61.6% and 20.3%), 5A and 5B (51.8% and 15.9%), and 6A and 6B (36.2% and 16.7%), 7A (15.9%), 8A (22.5%) and 9A and 9B (47.8% and 19.6%) as shown on Table 2.

**Table 2:** Item performance of the SCQ in a sample of acute hospital staff in Australia

Item	Mean	SD	skewness	Kurtosis	Floor/ceiling effect %	Corrected item-total correlation	Cronbach’s alpha if item deleted	Mean of index scores	SD of index scores	Corrected item-total correlation index scores	Cronbach’s alpha if item deleted on index scores	Factor loading index scores
1A How often do you lack the time to provide the care the patient needs?	3.68	1.50	− 1.065	.126	4.8/39.4	.54	.81	13.95	7.40	.60	.82	.706
1B Does this give you a troubled conscience?	3.57	1.34	− 1.278	1.501	2.2/10.5	.50	.82					
2A Are you ever forced to provide care that feels wrong?	1.33	1.43	.779	− .545	40.9/2.9	.55	.81	5.21	6.32	.56	.82	.669
2B Does this give you a troubled conscience?	2.53	1.92	− .178	− 1.587	14.9/5.4	.47	.83					
3A Do you ever have to deal with incompatible demands in your work?	2.94	1.62	− .420	− 1.014	10.9/17.4	.61	.80	10.48	7.92	.63	.81	.729
3B Does this give you a troubled conscience?	3.15	1.50	− .714	− .468	3.3/6.5	.57	.82					
4A Do you ever see patients being insulted and/or injured?	0.60	.95	1.699	2.651	61.6/0.4	.34	.83	2.16	3.74	.44	.83	.551
4B Does this give you a troubled conscience?	2.13	2.06	.484	− 1.530	20.3/9.1	.55	.82					
5A Do you ever find yourself avoiding patients or family members who need help or support?	1.02	1.41	1.095	− .030	51.8/1.4	.51	.81	3.63	5.60	.38	.82	.681
5B Does this give you a troubled conscience?	2.18	1.73	.223	− 1.595	15.9/5.1	.59	.81					
6A Is your private life ever so demanding that you don’t have the energy to devote yourself to your work as you would like?	1.18	1.29	1.057	1.602	36.2/2.5	.51	.81	3.68	5.14	.30	.83	.579
6B Does this give you a troubled conscience?	2.06	1.93	.233	− 1.411	16.7/2.5	.58	.81					
7A Is your work in healthcare ever so demanding that you don’t have the energy to devote yourself to your family as you would like?	2.45	1.62	− .130	− 1.263	15.9/9.1	.66	.80	9.52	7.89	.47	.81	.755
7B Does this give you a troubled conscience?	3.25	1.69	− .668	− .961	4.4/9.4	.61	.81					
8A Do you ever feel that you cannot live up to others’ expectations of your work?	1.89	1.55	.361	− 1.062	22.5/5.4	.57	.81	6.68	6.77	.43	.82	.670
8B Does this give you a troubled conscience?	2.75	1.74	− .318	− 1.292	8.7/5.8	.57	.81					
9A Do you ever lower your aspirations to provide good care?	1.08	1.40	1.112	.346	47.8/3.3	.49	.82	3.55	5.69	.36	.83	.604
9B Does this give you a troubled conscience?	1.95	1.81	.394	.305	19.6/4.3	.49	.82					

***SD*** standard deviations Index scores = Item A multiplied by Item B

### Reliability

All individual items met the cut-off criteria for item-total correlations above 0.3 as shown in Table 2. Further evidence of satisfactory internal consistency reliability was indicated by a total Cronbach’s alpha of 0.84, not being increased by deleting any of the items. The results were also consistent if the Part A and Part B questions were measured for reliability separately or as combined index scores (Part A multiplied by Part B).

### Dimensionality

Exploratory factor analysis (EFA) was conducted on the index scores (Part A multiplied by Part B) as proposed by Glasberg et al. [3]. All SCQ items had correlations within the recommended range of 0.30 to 0.70 with at least one other item. The Kaiser–Meyer–Olkin (KMO) was 0.84 and Bartlett’s test of sphericity was significant (p < 0.001). The Kaiser criteria of an eigenvalue > 1, the Cattel scree test and parallel analysis yielded one single latent factor which explained 44% of the total variance. All items met the criterion of communalities exceeding 0.3 in the principle component analysis (PCA). The unrotated factor matrix loadings were greater than 0.55 (Table 2). When maximum likelihood extraction and principle axis factoring was performed, a single factor structure was also retained with adequate factor loadings.

**Fig. 1:**
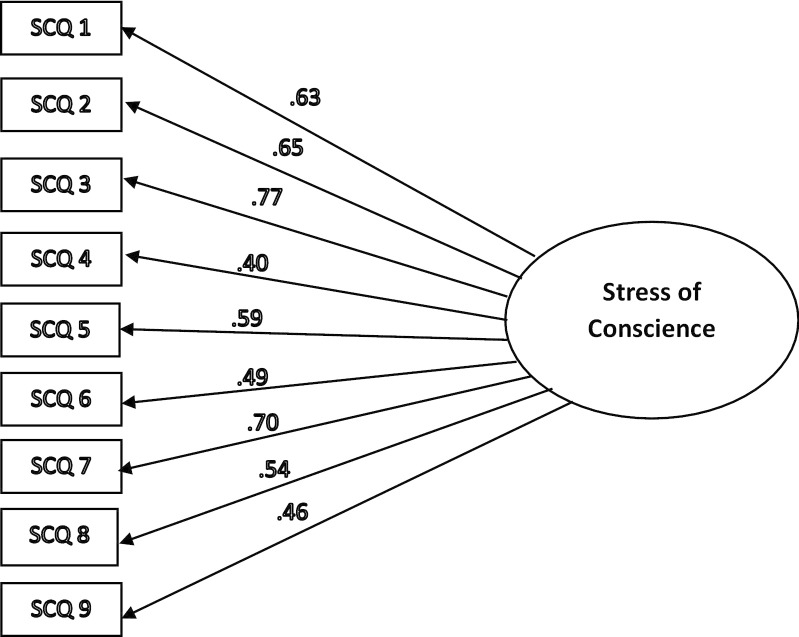
Confirmatory Factor Analysis (CFA) of the one-factor structure proposed in this study using AMOS software. SCQ = Stress of Conscience Questionnaire. SCQ 1 to 9 = Stress of Conscience Questionnaire Index Score Items (Part A multiply by Part B of each item). CFA identifying variance was 1 and factor loading cut-off was set > 0.4

**Fig. 2:**
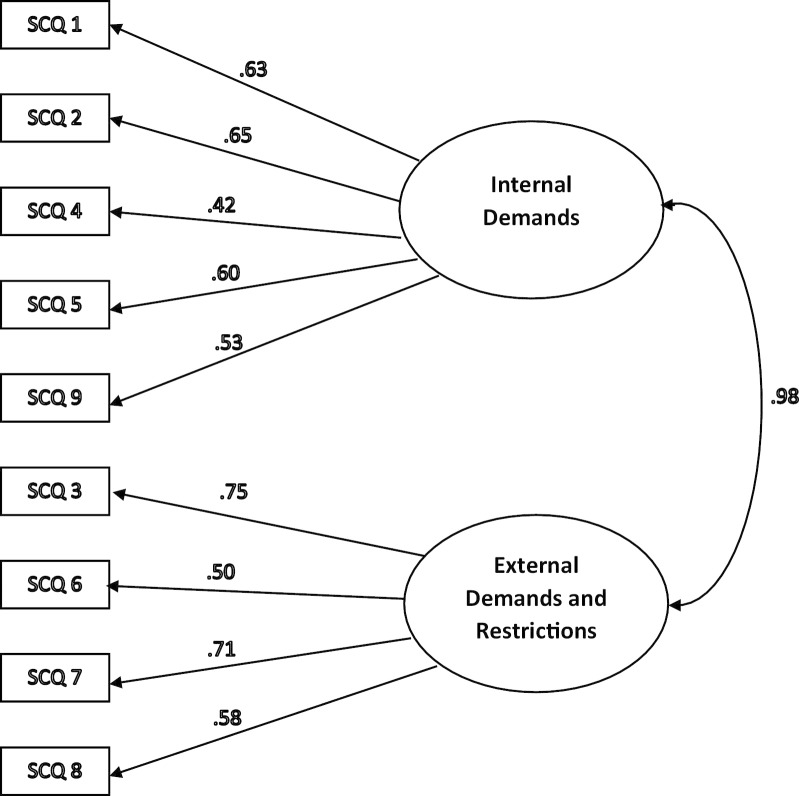
Confirmatory Factor Analysis (CFA) of the two-factor structure proposed by Glasberg et al. (2006) using AMOS software. SCQ = Stress of Conscience Questionnaire. SCQ 1 to 9 = Stress of Conscience Questionnaire Index Score Items (Part A multiply by Part B of each item). CFA identifying variance was 1 and factor loading cut-off was set > 0.4

The single latent factor was compared with the two latent factors proposed by Glasberg et al. (2006) (i.e. Factor I = Internal Demands and Factor II = External Demands and Restrictions) as a one-factor and a two-factor model. The one‐factor model was associated with good model fit (CMIN/DF = 1.340; P-value = 0.146; CFI = 0.990; AGFI = 0.948; RMSEA = 0.037 and PCLOSE = 0.704) as shown in Table 3. Factor loadings ranged from 0.40 to 0.77 as shown in Fig. 1. In contrast, the two‐factor model fit indicated that some fit indices were not adequate (CMIN/DF = 3.521; P-value = 0.000; CFI = 0.910; AGFI = 0.871; RMSEA = 0.100 and PCLOSE = 0.000). Factor loadings for the two‐factor model ranged from 0.42 to 0.75 and two latent factors also closely correlated, which suggests a lack of distinct factors as displayed in Fig. 2. When the two‐factor model was compared to the one‐factor model, the latter received the lowest AIC score (AIC = 123.768) and the chi-square test was not significant (P-value = 0.146), which also supported the one- factor model as better fitting the data.

**Table 3:** Model fit summary

Model	CMIN/DF	AGFI	CFI	RMSEA	PCLOSE	CMIN Chi-square value	AIC
Reference values *Jackson *et al*. 2009* *Hair *et al*. 2010*	< 3 Good < 5 permissible	> 0.80	> 0.95 great > 0.90 traditional > 0.80 permissible	< 0.05 good 0.5–0.10 moderate > 0.1 bad	> 0.05	P-value > 0.05	The model with a lower value demonstrates better model fit
1-Factor Model	1.340	.948	.990	.037	.704	53.768 P-value = .146	123.768
2-Factors Model	3.521	.871	.910	.100	.000	80.982 P-value = .000	142.81

## Discussion

Exploration of the psychometric properties and dimensionality of the Stress of Conscience Questionnaire (SCQ), based on a sample of health professionals working in a tertiary-level Australian hospital, indicated satisfactory reliability and variability estimates. Also, the scale was found to be unidimensional as one single latent factor was confirmed. This suggests SCQ results can be aggregated, interpreted, and communicated as one summative score aggregating all individual 9-Items without the use of any subscales.

Although most SCQ items showed adequate variability, Item 1A and item 3A had a ceiling effect and a few other items showed a floor effect, indicating that more than 15% of results were aggregating at the top or bottom scoring alternative. The limited variability among these items could be problematic if the SCQ is used to assess variance over time or in pre and post interventions studies [24], and it remains unknown if this is due to data characteristics of this study or shortcomings in the questionnaire. Further studies would be valuable to explore the variability of these items. Higher mean scores obtained for Items 1 ‘*do you often lack time to provide the care?* Item 3 ‘*Do you ever have to deal with incompatible demands?*’ and Item 7 ‘*Does your work affect your private life?*’ were consistent with previous studies [12, 13]. Although Ahlin et al. [4] suggested removing Item 6 ‘*Is your private life ever so demanding that you don’t have the energy to devote yourself to your work as you would like?*’, this study demonstrated that the item should be retained due to having an adequate correlation with other items and a factor loading of 0.58. The Cronbach’s alpha of 0.84 and item-total correlations (ranging between 0.30 and 0.70) indicated that all items reliably measured a single underlying construct with acceptable homogeneity [24]. Reliability scores were stable both when Part A and Part B questions were treated separately or as index scores (Part A multiplied by Part B) as indicated in Table 2. Initial validation by Glasberg et al. [3] and subsequent revalidation by Ahlin et al. [4] also showed adequate internal consistency, indicating stability of the SCQ across different samples and settings.

This is the first study to explore and confirm unidimensionality of the SCQ in an English-speaking context, which adds further evidence and confidence for use of the Stress of Conscience Questionnaire. The results of the EFA yielded a single latent factor, which explained 44% of the total variance. Factor loading of each item was greater than 0.55 on the first extraction factor, meaning that all items were indicators of the latent factor, Stress of Conscience. However, the studies by Glasberg et al. [3], Ahlin et al. [4] and Saarnio et al. [17] retained two latent factors. Although these studies produced two stable latent factors, there were higher cross loading on both latent factors. The initial validation by Glasberg et al. [3] included item 1 in both latent factor solutions. In addition, the rotated two-factor solution by Ahlin et al. [4] was inconsistent with the theoretical interpretations proposed by Glasberg et al. [3]. According to Ahlin et al. [4], all items except for Item 6 had higher loadings on the first factor (all > 0.48) compared with the second factor in the unrotated solution, which indicated a unidimensional structure. Ahlin et al. [4] concluded that this outcome could be a result of the index scores, which equalizes stressors and troubled conscience (Part A multiply by Part B). The factor structure in this study meets the criteria for unidimensionality, confirming that calculations of the arithmetic mean, from the summation of all index scores, provides meaningful data for interpretation, comparison and communication of results. This was reinforced by the CFA which indicated that the one-factor structure had better model fit (CMIN/DF = 1.340; P-value = 0.146; CFI = 0.990; AGFI = 0.948; RMSEA = 0.037 and PCLOSE = 0.704) as compared to the two-factor model (CMIN/DF = 3.521; P-value = 0.000; CFI = 0.910; AGFI = 0.871; RMSEA = 0.100 and PCLOSE = 0.000). Although some model fit indices were also acceptable for the two-factor model, the latent factors were highly correlated, suggesting that they were not distinct factors. The one‐factor model also received the lowest AIC score (AIC = 123.768) and the chi-square test was not significant (P-value = 0.146), indicating that this was the most parsimonious model for the data analysed [25, 26]. Therefore, the results of this study indicate that the SCQ is best conceptualized as a unidimensional measure with a single latent factor.

There were some limitations of this study of importance to consider. The self-reported data may be liable to social desirability bias, and thus needs cautious interpretation. However, the data collection process was anonymous to encourage participants to be truthful. The cross-sectional and contextual location of the data implies cautious interpretation of the findings, and further data from other contexts and countries is needed. Results from reliability and dimensionality testing also need to be interpreted with caution, as the criteria for assessing goodness of fit are relative rather than absolute [25]. The sample consisted mainly of female nurses, which may limit generalizability for different genders and other professions. The stability of the single latent factor also needs to be assessed as the parallel analysis factor explained 44% of the total variance.

## Conclusion

The Stress of Conscience Questionnaire achieved satisfactory reliability and variability for assessing frequently encountered stressful situations, and the degree individual health professionals experience a troubled conscience in their workplace. The factor structure in this study met the criteria for unidimensionality, suggesting that a simple sum score of items is a feasible and reliable way forward to quantify and explore this phenomenon across countries, and different contexts. Highlighting and discussing ethical challenges is at core of healthcare, and the SCQ can be a significantly helpful tool for clinical managers in this process.

## Data Availability

The dataset analysed during this study is available from the corresponding author on reasonable request.

## Abbreviations

AGFI: Adjusted goodness of fit
CFI: Comparative Fit Index
CFA: Confirmatory factor analysis
EFA: Exploratory factor analysis
KMO: Kaiser–Meyer Olkin measure of sampling adequacy
RMSEA: Root mean square error of approximation
SCQ: Stress of Conscience Questionnaire
